# A comparison of presentation methods for conducting youth juries

**DOI:** 10.1371/journal.pone.0218770

**Published:** 2019-06-26

**Authors:** Liz Dowthwaite, Elvira Perez Vallejos, Ansgar Koene, Monica Cano, Virginia Portillo

**Affiliations:** 1 Horizon Digital Economy Research Institute, University of Nottingham, Nottingham, United Kingdom; 2 NIHR Biomedical Research Centre for Mental Health and Digital Technologies, University of Nottingham, Nottingham, United Kingdom; Middlesex University, UNITED KINGDOM

## Abstract

The 5Rights Youth Juries are an educational intervention to promote digital literacy by engaging participants (i.e. jurors) in a deliberative discussion around their digital rights. The main objective of these jury-styled focus groups is to encourage children and young people to identify online concerns and solutions with a view to developing recommendations for government policy-makers and industry chiefs. The methodology included a series of dramatized scenarios that encourage jurors to deliberate about their digital rights. This paper compares two formats for these scenarios: live actors and professionally recorded and edited videos of the same actors. Results failed to show any major differences between formats indicating the cost-effectiveness of the video-recorded format and the possibility for others to run the 5Rights Youth Juries with the support of an online open educational resource.

## Introduction

The 5Rights Youth Juries are similar to focus groups in which emphasis is given to the principles of deliberation [1–3]. They allow young people, generally teenagers, to receive and exchange information about their online experiences, to critically examine their digital rights, and to come to an agreement with a view to informing decision making as well as influencing policy-makers. The goal of this paper is to explore the possible outcome differences between the format presentations of scenarios showcased at the juries. It is hypothesised that scenarios presented via live drama are going to be more engaging than those presented via video format. Differences between formats could lead to differences in outcome measures (i.e. attitudinal change). The absence of differences between formats, however, would illustrate that the methodology used in the juries is robust across different presentation modes, and can be used effectively by educators and other stakeholders through an online open educational resource.

The evaluation and comparison of different format presentation needs further research to be more conclusive. Empirical evidence is scarce and the effectiveness of various types of educational programmes comparing drama and video show varying results. This study addresses this gap in assessing the factors which influence the effectiveness of presentation formats in the context of digital literacy.

### Digital literacy and digital rights

Programmes designed to increase digital literacy among children and young people (see [4] or [5] for a review) include approaches to increasing information and communication technology (ICT) skills (e.g. how to use a keyboard or search for information) and online safety [6]. Alternative and complementary approaches have focused more on developing a critical understanding of the digital world while building resilience and opportunities [6–10]. New recommendations focus more on how best to empower children as rights-holders to respect, protect, and fulfil the rights of the child in the digital environment [11–16]. This paper contributes to this emerging and under-researched area.

The juries are designed to explore and discuss five specific digital rights identified and put forward by the 5Rights Framework coalition (see www.5RightsFramework.com), which are:

- The right to remove, enabling young people to easily edit, delete, retract, correct and/or dispute content they have created and/or data that refers to them.
- The right to know, designed to increase transparency concerning how young people’s information is being used and who is holding and profiting from such information.
- The right to safety and support, promoting the same age-appropriate and compatible protection, care and support online as in the offline world.
- The right to be informed and make conscious choices, empowering children to recognize when their attention is being manipulated and empower them to disengage at will.
- The right to digital literacy, aimed at providing young people with the digital skills necessary for using, creating and critiquing digital technologies and giving them the tools for negotiating social norms.

A rights-based approach to media and information literacy is important because it provides a framework that is operationally directed to engaging young citizens on fundamental human rights to freedom of information, expression and education. From a human rights-based perspective, the 5Rights framework is a composite set of knowledge, skills, and attitudes “that enables and empowers citizens to competently and critically engage with media and information, in order for them to increase their individual autonomy and collective solidarity in society” [17].

In order to illustrate concrete examples for each of these digital rights, five scenarios describing online youth experiences were co-produced with a matched sample. Co-production ensures that scenarios (i.e., stimuli or prompts) represent real issues and experiences that young people can relate to. As a consequence, scenarios become idiosyncratic and sensitive to cultural differences, represent a specific and distinct point in time, and avoid universalistic terms.

### The use of vignettes in education

The use of dramatic scenarios builds upon the methodological research tradition of using vignettes as prompts to elicit reflective responses from participants. There is a long tradition of using vignettes within educational settings, and there is extensive evidence of the underlying social, cognitive and emotional processes associated with using applied drama for facilitating learning and development [18–21].

While the format of vignette presentation can vary, the aims and objectives are usually the same: to facilitate discussion, reflection, and deliberation amongst a group of young people (in this case, the jury) that may develop new attitudes, opinions, and interpretations about the topic of the vignettes (in this case their digital rights and therefore, the potential benefit and harm associated with specific online activities). The development and administration of vignettes should always protect the research participants, especially when presenting sensitive issues [22], providing a safe space for open discussion. Most often vignettes are short stories that are read aloud to participants. Some researchers have used film and music, while others have used interactive web content or live acting, deriving value from combining the stimulus of the vignette method with the liveness and indeterminacy of the applied drama/theatre-in-education tradition [23,24].

Vignettes have been used by researchers from a range of disciplines, including scholars studying public acceptance of mentally ill residents within a community [25], multicultural integration in neighbourhoods [26], the neglect and abuse of elderly people [27], and early onset dementia [28]. Vignettes have proved to be particularly useful in eliciting reflective responses from groups of young people. For example, [23] used them very successfully to research violence in residential children’s homes; [29] used vignettes as a way of talking to young rural Canadians about what they considered to be ‘risky activity’; [30] used them in their work with adolescent girls to explore their lived experiences of physical exercise; and [31] employed vignettes to explore children’s experiences of domestic abuse.

### Using vignettes to focus on attitudes to digital technology

While there is a considerable amount of literature that documents the efficacy of vignettes within educational settings [32], hardly any systematic research has focused on using vignettes to promote changes in attitude towards digital technologies. According to [33], there are three main problems that any digital literacy programme should address. The first is the participatory gap, which refers to inequalities in young people’s access not only to new media technology and the Internet, but also to the skills and content that are most beneficial. The second is the transparency problem, which highlights the lack of “a clear and plain language that the child can easily understand” which is now required in the UK by the General Data Protection Regulation (GDPR) [34], and the potential commercial interests that may influence online decisions. For example, [35] argue that the Terms and Conditions of most online platforms do not offer any form of meaningful transparency to their younger users, and consequently it has fuelled a sense of exclusions amongst many young people. This becomes apparent for example in advertising practices in online gaming, and the dangers of blending false or inaccurate information with facts. It is especially relevant to how children make sense of online resources, with [36] finding a lack of both knowledge and interest in assessing how information was produced. The third problem is how to encourage young people to become more reflective and responsible about their online behaviour, the choices they make online, and the potential impact their choices may have on others. This ethics challenge is linked to digital citizenship and relates to the content young people post online, the content they have access to (e.g., adult content such as pornography), and compliance with implicit/explicit online community rules (e.g., Terms & Conditions). Digital citizenship provides a framework to understand what it means, for a young person, to be a responsible citizen online; how to protect their own online rights as well as to respect others’ rights. In the UK, there are already a number of digital citizenship curricula available [37] that also include practical advice on how to actively disengage as well as engage with the digital world and contribute to make the online environment a force for good and one which empowers children and young people rather than entraps them. Digital citizenship provides a framework to understand what it means, for a young person, to be a responsible citizen online; how to protect their own online rights as well as to respect others’ rights. In the UK, there are already a number of digital citizenship curricula available [37] that also include practical advice on how to actively disengage as well as engage with the digital world and contribute to make the online environment a force for good and one which empowers children and young people rather than entrapping them.

These three problems are central themes developed and dramatized in the 5Rights Youth Juries, particularly in the Right to Agency, the Right to Know and the Right to Digital Literacy. The connection between the problems and the rights should promote among young citizens an expectation that media and information sources delivers content of technical, ethical and professional quality, thus acting as a catalyst for improvements in journalistic reporting, editorial organisation and the media system as a whole. For example, the Right to Know Illustrates a scenario about the kind of personal data that is regularly tracked and stored when people go online:

*“The scenario begins with two actors*, *one young person and the other playing the part of the Internet*. *The latter says to the former*, *‘I know you’re feeling quite negative about me*, *so what I was thinking was maybe we could chill out*, *spend some time together*, *you could get to know me…’ They decide to go shopping together*. *As they go into each shop*, *the young person becomes increasingly surprised by how much the sellers know about her*. *One already knows where she’s shopped before; another knows her shoe size; a third has stored her credit card number*. *Exasperated by all of this*, *she exclaims*, *‘This is ridiculous*. *How come all these people know where I live*, *know my details and keep sending me emails*?*’ ‘Well*, *we are sharing information*. *It’s useful’*, *responds the Internet*. *‘How many people know this stuff*?*’ asks the young person*. *‘Around 56*,*’ replies the Internet*.*”* [38] (page 21)

The 5Rights Framework addresses the transference of knowledge, skills and attitudes in several distinct areas; accessing media and information (technical skills), using media and information (content decoding skills), evaluating media content (ability to judge credibility, objectivity and accuracy of sources), creating content (critical attitude), when and how to interact/participate with others online, knowing how media works, and demanding media quality and rights.

Consequently, the juries were designed to promote social skills and cultural competencies through dialogue, collaboration, and discussion. They offer objective information about data privacy issues, a space for reflection on how media shapes perceptions of the world, and opportunities to develop critical analysis skills. The dilemmas or conflicts that the scenarios bring to life include an element of reflection on the negatives as well as the positives exhibited on the Internet. They also encourage young people to pool knowledge and reconcile conflicting information to form a coherent picture. The youth juries introduce a form of problem solving valuable in shaping all kind of relationships (e.g., knowledge, community, tools, etc.).

### Using dramatised scenes in vignettes

The addition of dramatised scenes performed live during the juries added a realistic dimension to the deliberation process and served to highlight key themes and issues by bringing them to life and stimulating discussions. This could be considered a form of simulation, encouraging young people to interpret and construct models of real-world processes. The scenes are highly dynamic, allowing space for improvisation and interaction, so that young people can formulate hypotheses of ‘what is going to happen next’, test different variables in real time, and modify or refine their interpretation of the ‘real world’, while also being engaged in a process of modelling (i.e., learning that takes place in a social context through observation). It is well known that students learn more through direct observation and experimentation than simply by reading text books, or listening in the classroom setting [39]. Simulations not only broaden the kinds of experiences students may have but bring the capacity to understand problems from multiple perspectives, and to assimilate and respond to new information.

There is always an ongoing debate between the effectiveness of screen vs. theatre formats. Empirical evidence is scarce and some authors argued that the assessment of drama is especially problematic due to its multi-dimensional nature [40,41]. The effectiveness of various types of educational programmes comparing drama and video show varying results. For example, [42] compared the effectiveness of educational animated videos and drama in increasing the level of knowledge in 9–11 year old children about epilepsy and decreasing epilepsy-related stigma. Their results showed that while both groups achieved a significantly higher score than the control group on knowledge of epilepsy and attitudes towards children with this disease, the video was more effective than drama in improving knowledge of epilepsy. On the other hand, there was no significant difference between the two kinds of intervention regarding attitudes towards children with this disease. Another study comparing two disordered eating prevention programs (drama vs multimedia) showed that drama was more effective in changing cognitions than multimedia and the interactive program [43]. A great deal of future research is necessary to determine the factors influencing the effectiveness of different types of format presentations. This study addresses this gap in the context of digital literacy.

While live acting adds an element of excitement, the high costs and complex logistics involved may impede wider dissemination of such programmes, and consequently minimise participation. Video recordings are a plausible format for disseminating among secondary schools, allowing 5Rights Youth Juries to be easily recreated, shared and delivered within both drama and IT school departments. This study used the results from pre/post jury surveys to empirically test whether the two different presentation modes elicited different responses to digital technology. If the method of presentation does not affect the opinions of the young people, there should be no differences between the two waves of juries unless they existed prior to the sessions.

## Method

### Participants

Young people aged between 11 and 20 were recruited to take part in one of a series of 5Rights youth juries taking place in Nottingham, Leeds, and London. They were recruited by the SMH Foundation based in London and the CaSMa (Citizen-centric approaches to social media analysis) research team based in Nottingham. All participants filled out registration and consent forms and, if under 16, also needed to have the signed consent of a named teacher, youth worker, parent, or guardian. Upon completion, participants received a £10 shopping voucher as thanks. In total, 216 participants took part in the youth juries: 107 attended wave 1 across 9 juries that included live-action versions of the scenarios, and 109 attended wave 2 in 7 juries that involved video-recorded versions of the same scenarios. The average age of both groups was 15 years old, and the gender was roughly even in both groups (55% female in the live-action group, and 56% female in the video group). Respondents in both groups predominantly used digital devices, like phones, game consoles and PCs for playing games, using apps, and accessing websites, several times each day (94% in the live-action group, and 96% in the video group). The similar demographics minimise any potential biases between groups. Whilst it might be reasonably expected that those young people who signed up to take part in the juries had an existing interest in the topic, this would be the same for both groups and therefore would not cause a problem with comparing the responses between groups.

### Materials and procedure

The study was approved by the Ethics Review Board for the Department of Computer Science at The University of Nottingham.

The youth jury methodology is described in detail elsewhere [38] as well as the qualitative results derived from the deliberation process from wave 1 of the juries [44]. This paper focuses only on the quantitative data collected via surveys completed before and after the juries (see S1 Appendix). All 16 of the youth jury sessions took the same format over approximately 2 hours including refreshment breaks. Where possible, the layout of the room formed a horseshoe so that all participants could face the actors or the screen. Each session involved roughly 12 young people and all sessions were video- and audio-recorded for later transcription. Participants were first asked to complete a pre-session survey consisting of 10 brief multiple choice questions which aimed to gauge the level of knowledge participants had going into the sessions, and their existing opinions about issues of relevance. These include their current use of digital devices and online services, their knowledge of removing content and online safety, who regulates the digital world, and how much say they felt they had in how the digital world works.

Participants were then presented with a series of vignettes and encouraged to discuss the issues that each scenario raised as well as being asked for recommendations relating to the problem presented to them. The juries were all moderated by an experienced facilitator, an adult previously unknown to the participants and who was not presented as an authority figure. The facilitator made sure all jurors had the chance to be heard, with all experiences, viewpoints, and recommendations seen as valid and respected by all members of the jury; the sessions were guided in a way that was not leading or instructive so as not to prescribe opinions. Discussions took the form of a deliberation after each vignette was presented, with participants encouraged to break-out into smaller groups of 6 to 8 participants for 15 to 20 minutes of discussion. This allowed participants to share opinions with an emphasis that there were no right or wrong answers.

There were five vignettes, of 2 to 3 minutes in length. Each related to one of the fundamental rights put forward in the 5Rights Framework [38]: the right to know, the right to remove, the right to safety and support, the right to make informed and conscious decisions, and the right to digital literacy. In each, one actor played the part of the Internet as an embodied human being, with another playing the part of the internet user, and others acting as websites that they may encounter. The scenarios were co-developed over 2 weeks with an applied drama practitioner and drama students at the University of Leeds (School of Media and Communication). They were designed to be discursive rather than prescriptive, to prompt responses and recommendations; scenes were open-ended and could be resolved in a number of different ways. As such, the vignettes presented scenarios about: the kind of personal data that is regularly tracked and stored when people go online; the desire to delete online content that may be embarrassing or inconvenient; unhealthy dependence upon digital communication technologies; and how online networks can cause young people to feel excluded or anxious and therefore affect their self-esteem. The same vignettes were presented in each youth jury, however in wave 1 these were performed live by actors, and in wave 2, the participants were shown pre-recorded videos.

The sessions ended with a post-session survey. This survey consisted of 3 brief multiple choice questions about how much the participants felt they had learnt from the session, followed by a series of 11 statements designed to measure opinion on the issues raised; 6 statements were scored on a Likert scale from 1 (agree very little) to 10 (agree very much), and 5 were scored on a Likert scale from 1 (applies to me very little) to 10 (applies to me very much). Statements covered similar issues to those from the pre-survey, including removal of content, online safety, regulation and power, and perceptions surrounding agency in how the digital world works. The statements, and the multiple choice questions in the pre-session survey, were designed to align with the principles of the 5Rights Framework and to determine how knowledge, attitudes, and opinions change when people are given a chance to deliberate on the issues. For example, the vignette based on the Right to Remove relates to post-session questions about how easy it should be to remove content, the Right to Safety and Support to questions of online safety, and the Right to make Informed Decisions to questions about agency.

This paper focuses on comparing the two presentation modes, aiming to identify whether the method of presentation (live versus recorded) affected participants’ opinions during the juries. To do this statistical analysis (Chi-square and t-tests [45]) was carried out on the responses to pre- and post- session surveys between the juries using live actors and those using recorded vignettes. This analysis allows for comparison of how much each type of jury affected the attitudes and opinions of the participants. A lack of differences between responses shows that the presentation method does not affect deliberation of the issues highlighted in the 5Rights Framework.

## Results

This section compares responses from the pre- and post-surveys, across both groups of jury attendants, to identify any differences in response patterns or change in opinion.

### Pre-existing differences between the groups

**Fig 1:**
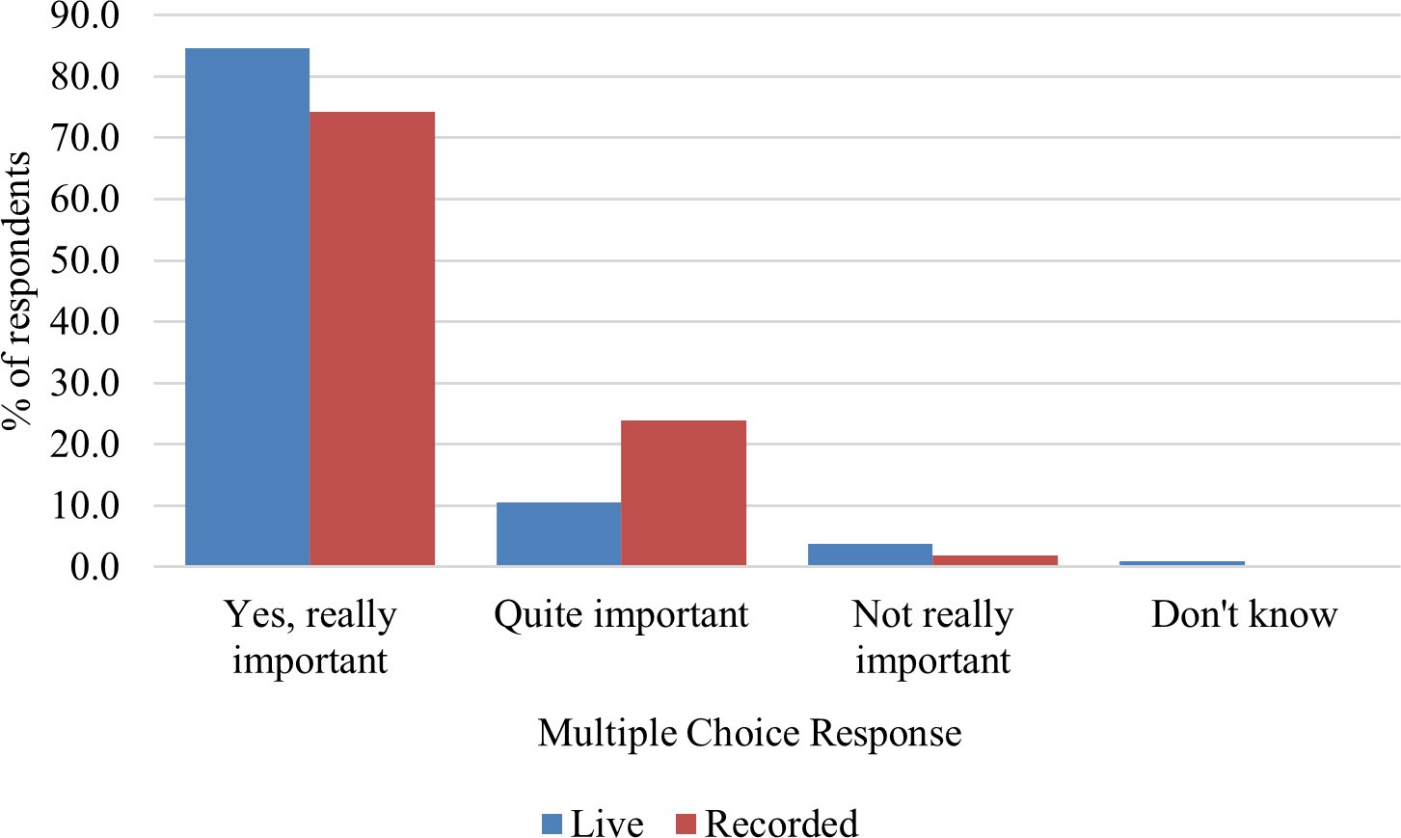
Responses to “Do you think that it’s important for people to know how to remove stuff that they put up on a site or app?”.

According to the pre-session survey, around three quarters of respondents in both groups felt that they knew how to remove content that they put up on a website or on an app, and that this knowledge was highly important. There was no difference between groups in their perceived knowledge, *X*^2^ (3, 216) = 6.54, NS. There was however more uncertainty amongst the recorded group about the importance of knowing, with 24% feeling it was only quite important to have this information. This difference is significant, *X*^2^ (3, 213) = 7.92, p < 0.05, so the live group felt it was more important, as shown in Fig 1. Over a quarter of people in both groups also did not know where they could go for help if they were stressed or upset online (28% live, 29% recorded), and the difference between groups was not significant *X*^2^ (1, 213) = 0.06, NS.

In terms of who monitors the digital world, making it safe and supporting for users, respondents in both groups predominantly thought it was the big tech companies (51% both live and recorded), and the difference between groups was not significant *X*^2^ (3, 210) = 2.04, NS. The most common choice of both groups was that such companies had too much power over their lives (40% live, 45% recorded). More people in the live group felt they had the right amount of power (30% live versus 22% recorded) and more in the recorded group felt they only had a little power (27% recorded versus 20% live). However, the differences between groups were not significant, *X*^2^ (3, 210) = 3.57, NS.

**Fig 2:**
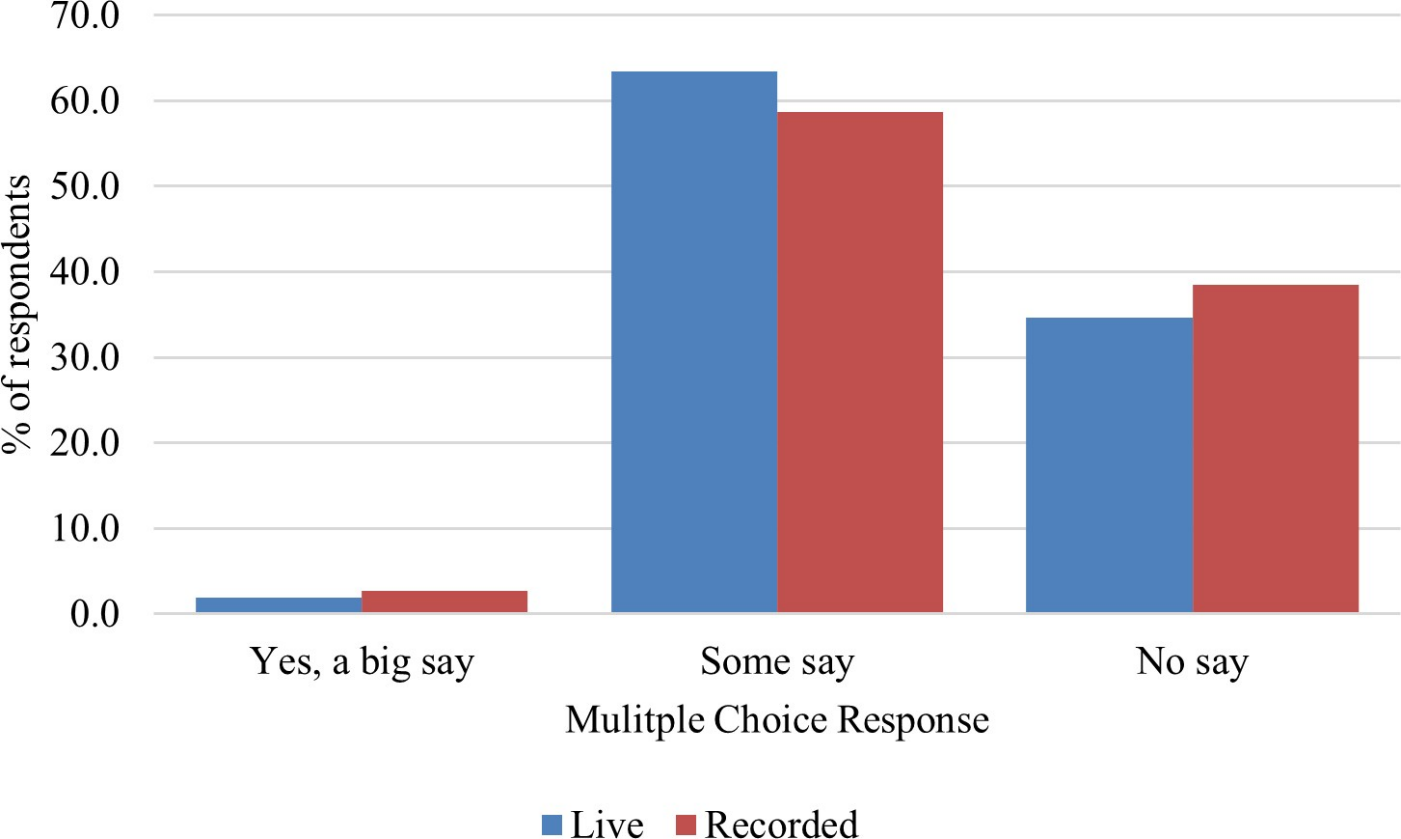
Responses to “Do you think that you have any say at all in how the digital world works?”.

Most people in both groups felt that they had some say in how the digital world works (64% in the live group and 59% in the recorded); very few felt that they had a large say, and a substantial number in both groups felt they had no say, as shown in Fig 2; there was no significant difference between the groups, *X*^2^ (2, 213) = 0.58, NS. The majority of both groups felt they should have more say in their digital world (60% of the live group and 55% of the recorded group), although around a quarter of both groups were either ‘not bothered’ or didn’t know, and once again the difference was not significant, *X*^2^ (3, 213) = 1.14, NS.

In summary, differences between the two groups (live and recorded) before the juries were minimal, with the only significant difference being that the live group thought it was more important to know how to remove content that they put up on a website or on an app than the recorded group did.

### Post-Survey changes

After the session, most people in both groups felt that they had learnt at least something new about how the Internet works (92% of the live group and 94% of the recorded group). They also predominantly felt that they had learnt at least something about how the Internet affects their lives (86% of the live group and 89% of the recorded group). Finally, they overwhelmingly felt that they had come up with at least a few ideas about how the Internet could be made better for people like them (98% of the live group and 95% of the recorded group). In addition to learning new things and creating new ideas, a large number of young people in both groups felt that they had changed their minds about how the Internet and other digital technologies should work (50% of the live group and 41% of the recorded group). The difference in the pattern of responses from both groups is not significant (t(201) = 0.775, NS).

**Fig 3:**
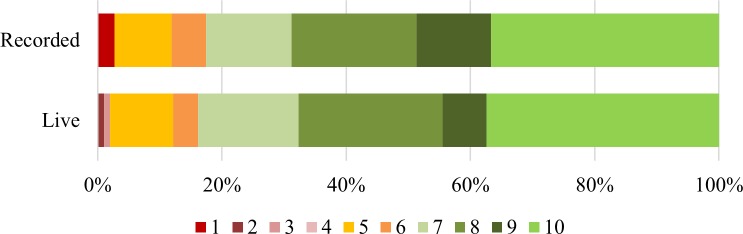
Responses to the statement “It should be made easier for people to remove digital content about themselves”.

As the following questions were scored on a 10-point scale, a score of 7 or above indicates agreement, and a score of 4 or below indicates disagreement. A score of 5 or 6 was considered neutral. Participants felt that “it should be made easier for people to remove digital content about themselves that’s embarrassing” (84% live, 83% recorded), see Fig 3. The average response in both groups was high (8). There was no significant difference between the pattern of responses in the two groups, shown by an independent samples t-test (t(206) = 0.089, NS). This response is roughly equivalent to the answers collected before the sessions about the importance of being able to remove content, where there was a difference between the two groups.

Participants in both groups overwhelmingly felt that “there should be a trustworthy place for young people to go if they are stressed or upset by things that happens to them on line” (93% of live and 90% of recorded group); the average score for both groups was 9. Over three quarters of each group did not agree that “it’s OK for websites to pass on details to other businesses or services about people who visit them” (79% of the live group and 76% of the recorded group); the average score for both groups was 3. The differences in the patterns of responses to each of these statements were not significant (t(206) = 1.285, NS and t(186.019) = -0.479, NS, respectively). This relates to the question about knowing where to go when stressed online that was asked before the juries, in which there was also no difference between the groups.

**Fig 4:**
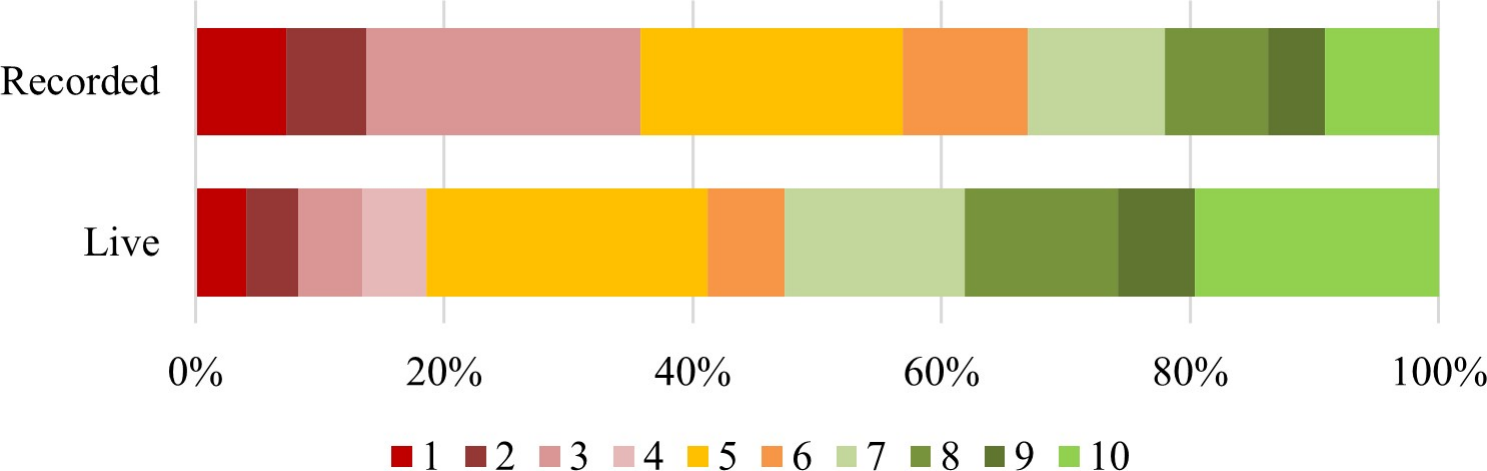
Responses to the statement “I’m confident that I can influence the way that digital technologies work for young people”.

**Fig 5:**
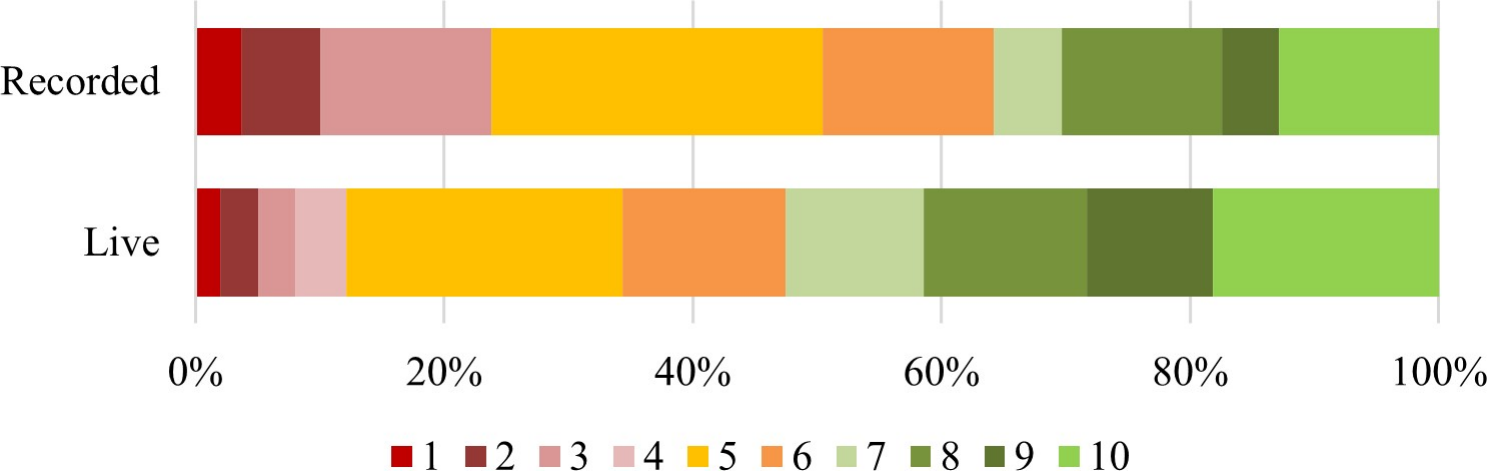
Responses to the statement “When I use digital technologies, I’m in charge of what happens to me”.

In the live group, after the sessions most people were “confident that [they] can influence the way that digital technologies work for young people” (53%, and an average score of 7), whilst the recorded group were less confident (just 33% responded positively and there was an average score of 5). The difference in response patterns is significant (t(204) = 3.345, p < 0.01). The main difference can be accounted for in the negative responses (19% of live compared to 36% of recorded), as shown in Fig 4. The statement “when I use digital technologies, I’m in charge of what happens to me” showed that whilst most people in the live group felt that they were at least somewhat in charge with an average score of 7, the recorded group were less sure with an average of 6. The difference between patterns of responses was significant (t(206) = 2.763, p < 0.01). As seen in Fig 5, 53% of the live group responded positively compared to 36% of the recorded group; 24% of the recorded group responded negatively compared to just 12% of the live group.

**Fig 6:**
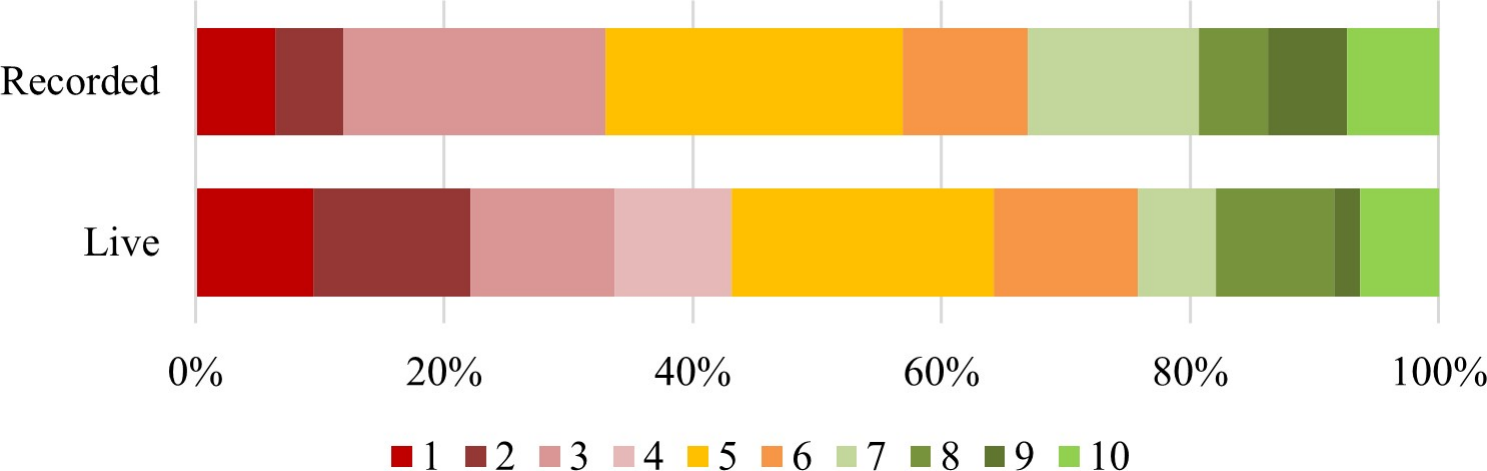
Responses to the statement “Nobody is going to listen to what young people say about the Internet they want”.

People in both groups tended to, at least partially, disagree that “nobody is going to listen to what young people say about the Internet they want”, as shown by Fig 6. Here, a low score was recorded for 43% of the live group and 33% of the recorded group, with a third of both groups responding neutrally (33% of live and 34% of recorded group), showing again that the recorded group remained less confident about their own power online. An average response of 5, or neutral, was recorded by both groups, and the difference in response patterns is non-significant (t(202) = 1.353, NS). These three statements relate to the pre-survey question about how much say they thought they had, which showed no differences between the groups.

There was an increase in the number of people in both groups who thought that “12–18 year olds should have a bigger say in how digital services and technologies are run” (73% of the live group and 64% of the recorded group) and that “when [they] use digital technologies, [they’d] like to be more in charge of what happens to [them]” (90% of the live group and 78% of the recorded group). There was an average score of 8 for the first item and 5 for the second item from both groups, with no significant difference in response patterns for either (t(206) = 1.588, NS, and t(203) = 1.265, NS, respectively). This relates to a similar question before the juries, where participants were asked if they wanted more say in how the digital world works, and in which there was also no difference between the groups.

## Discussion

This paper compares the survey results of two waves of 5Rights Youth Juries. The main difference between these two waves of juries is the format in which the scenarios were presented. While in wave 1 (Live) a group of young drama students from Leeds University developed and enacted the previously co-produced scripts, in wave 2 (Recorded) a professional video production company filmed and edit the dramatized scenarios. The results show that for most measures there were no differences in opinions between recorded and live scenarios. There are some differences, however, on the importance of removing content, and having a say in how the digital world works. These differences are discussed in more detail below.

Before the juries, both groups felt they mostly knew how to remove content they put online, but more participants who went on to see the recorded action version of the scenarios felt that it was only quite important to know how to do so. After the juries, both groups felt equally strongly that it should be easier for people to remove digital content about themselves, scoring the item highly. This suggests that participants in the recorded group who originally felt it was only quite important to know may have decided it was actually very important, whilst the Live group already held that opinion and were unable to increase their reported level of importance.

Before the juries, there was no difference between groups in how much say they thought they had in how the digital world works. Over half of both groups felt they had some say, and around a third of both felt they had no say. Very few felt they had a large say. The post-survey had three statements related to this question, two of which showed a significant difference between groups. For “I’m confident that I can influence the way that digital technologies work for young people” and “when I use digital technologies, I’m in charge of what happens to me”, people in the recorded group gave a neutral response on average whilst the live group agreed with both statements. The direction of responses for both of these items did however follow the pattern of the pre-survey question, with the recorded group appearing more sceptical in general, which may go some way to explaining this difference. The third statement “nobody is going to listen to what young people say about the Internet they want” was responded to neutrally on average by both groups, with many in both groups also disagreeing. However overall, both groups predominantly left the sessions feeling they had more say in their online lives than they did before.

There are some differences between the live and recorded juries that could be influencing the results of this comparison. First, the first wave of juries were held in three UK cities (London, Leeds and Nottingham) and the participants were recruited mainly from youth clubs and youth community groups. For the recorded juries, participants were recruited only from Nottingham city via youth community groups but also via secondary schools. While the live juries took place always outside the educational environment of the participants (i.e., university or community meeting rooms), at least one third of the recorded juries took place at their schools, usually as part of their ICT curriculum. The different environments could have influenced the pattern of responses, indicating that when participants are outside their schools their responses are more assertive, because they are feeling more confident and in charge. Numerous studies (see [46], for a review) have found that participation in extracurricular activities is associated with increased achievement outcomes [39,47–50], reduced dropout [51], and higher educational attainment [52], even after factoring in differences in student background. These results indicate that young people’s time use and activity participation outside regular school hours have been linked consistently to a number of positive academic outcomes and therefore, it is advisable that future juries–if possible–are conducted outside school and ideally outside school hours (e.g., weekends).

A second aspect that could have influenced the patterns of responses was the style and experience of the facilitators. Even though the style was similar, the facilitator of the live juries had more years of experience working with children and young people than the researcher that facilitated the recorded juries. A more experienced facilitator could have influenced the pattern of responses inspiring more jurors to have ‘a stronger say in their digital world’. Similarly, the less experienced facilitator in the recorded juries could have inadvertently introduced more negativity either within the examples or facts introduced during the sessions. There are other, more subtle, biases that could affect the outcome or direction of the discussion including body language and facial expressions that could favour one position or argument more than another. The research team was aware of this limitation and special attention was given to minimise undesirable effects that could contaminate the jurors’ arguments and counterarguments.

An important part of the youth jury method was the co-creation of materials. There are some limitations to co-production, for example some authors have suggested that it may not properly represent young people’s voices and simply channel them into pre-existing adult agendas [53,54]. However, the opportunities in this area outweigh the problems. It is important that young people are consulted on materials that are designed to communicate with them, and once these materials are created they are very effective. Co-creation and the youth jury method gives young people a voice in what they wish to focus on, allowing them to highlight the areas they feel are important, and things they care about, as well as enabling critical thinking. It is also important to mention that the topics discussed among jurors varied enormously from jury to jury. The juries were noisy and discursive, and jurors became aware that they were engaged in a process of collective judgement, one that called for both candour and compromise. In seeking to create a space in which children and young people could respectfully express their personal experiences, viewpoints and recommendations, jurors were encouraged to discuss how, or if, the scenarios related to their own experiences. At the same time they worked as a group to think through a set of recommendations that adults in general, and policy-makers and the digital industry in particular, would feel compelled to take seriously. The individual experiences discussed were personal; this variability could have influenced the different recommendation responses between juries. Further comparison of the qualitative content of the jury discussions may be useful to see if there is a systematic difference between the live and recorded wave discussions that differs from the differences between juries within each wave.

Most participants in both presentation groups left the sessions feeling that they had learnt something new about how the internet works and about how it affects their lives. They also overwhelmingly felt they had come up with some ideas for how the internet could be made better for people like them. This suggests that the sessions were effective at increasing both the knowledge and perceived self-efficacy of young people when they are online, regardless of the presentation method. Digital literacy is increasingly important to the ever-more-connected world that children and young people grow up in and the Youth Jury method is effective at increasing this literacy, especially in the area of their digital rights. Educators and professionals who work with young people on a regular basis, and are interested in promoting digital citizenship and literacy, have also recognised value in the Youth Jury approach [55]. As such, this paper contributes to the assessment and development of effective programmes for increasing critical understanding of the digital world, and empowering children as rights-holders with the capacity to make their own decisions about the use of online technologies.

As discussed in the Introduction, there have been mixed results of media presentation in different contexts, but in the context of digital literacy, this study shows that different presentation methods achieved similar changes in perception and attitude. Digital resources are a valid and cost-effective way of engaging young people in discussion and encouraging them to consider differing attitudes. These methods can be applied to a wide range of topics across digital literacy and citizenship.

Having shown that video presentation of the vignettes is no different to live performance, these co-created videos have been made available to anyone who would like to run similar Youth Jurys discussing digital rights. Taking into consideration the different demographics of young people both in schools and at extra-curricular clubs, an Open Educational Resource (OER)[56–58] has been co-designed with a group of educators. The OER enables educators and youth leaders to feel confident in facilitating a jury with their different cohorts, whilst also allowing them to adapt the juries to suit their needs. This OER, available at http://oer.horizon.ac.uk/5rights-youth-juries/, is distributed under a Creative Commons license, and includes the videos used in this study as well as other useful materials. It also contains a video which illustrates how the scenarios were developed and presented, and all the instructions and materials required to engage young people in meaningful discussions about their digital rights. The aim of the OER is to increase the awareness and understanding of young people with regards to their rights when interacting with their digital world, and this form of presentation enables engagement with a wide range of people from different backgrounds to benefit from the method.

## Conclusion

This paper compared the results from a pre-/post-jury survey of two cohorts of young people that took part in the 5Rights Youth Juries using different presentation methods. While in the first cohort live acting was used to enact the scenarios, the second cohort was presented with professional video recordings of those same performances. Overall, the results showed that children and young peoples’ attitudes towards technologies were not significantly different depending on presentation mode. This shows that different methods of media presentation, particularly digital resources, are a valid, engaging, and cost-effective method for engaging young people in discussions of their digital rights. Differences identified in this paper can be explained by geographical location, facilitator experience, and environment. As these methods get used under many different circumstances and with different groups, these differences are to be expected, but the methods of engaging with young people using different forms of media use do not change the efficacy of the youth jury method.

An important part of the youth jury method was the co-creation of materials. This paper has also shown that materials created in consultation with young people are very effective. Through co-creation, young people are able to highlight the areas they feel are important, and things they care about. As previously stated, video presentation is cost-effective and as such allows researchers and teachers to reach a wide audience from different backgrounds.

Based on the results of all of the 5Rights Youth Juries an Open Educational Resource has been developed by researchers and educators to allow others to run such sessions with groups of young people. This paper has shown that overall juries run with the OER will be just as effective in stimulating discussion and promoting critical thinking and changes in attitudes towards the use of digital technologies, and raising awareness and knowledge of the online world. Future work will look at other methods for engaging young people and others in discussion of the digital world. The young jury method will be expanded to include co-creation of interactive activities and games aimed at increasing understanding; the approach (including co-creation of materials) will also be used to engage with older adults on similar issues. This group is another under-represented group in discussion of the internet and digital technologies, and their views should be included in considerations of digital literacy programmes. The research will also lead to the development of further OERs to allow these effective methods to be used by others with similar interests; finally, it is hoped that the methods will be used and researched in different contexts to contribute to the field of media presentation methods.

## Supporting information

S1 Appendix Pre- and Post- session questionnaires. Blank versions of the questionnaires provided before and after the jury sessions, the results of which are the basis of this paper.
(PDF)
