# Supplementary material for: A comparison of presentation methods for conducting youth juries
Source: PLoS One. 2019 Jun 26;14(6):e0218770. doi: 10.1371/journal.pone.0218770 (PMC6594631; doi:10.1371/journal.pone.0218770)
Supplement: S1 Appendix — Blank versions of the questionnaires provided before and after the jury sessions, the results of which are the basis of this paper. (PDF) [file pone.0218770.s001.pdf]

Youth Jury Pre-Session Survey

Age

Gender

1. How often do you usually use a digital device like your phone, digital game, app or website?

a) Several times a day
b) Once a day
c) Once every few days
d) Once every few weeks
e) Never

2. Do you know how to remove stuff that you put up on a site or app – like a photo, a tag, a comment or anything else?

a) Yes
b) Sometimes
c) No
d) Not sure

3. Do you think that it's important for people to know how to remove stuff that they put up on a site or app – like a photo, a tag, a comment or anything else?

a) Yes, really important
b) Quite important
c) Not really important
d) Don't know

4. Do you know where to go if you feel stressed or upset in any way when you're online?

a) Yes
b) No

5. Who makes sure that the internet and digital world is safe and supportive for users?

a) The police
b) Nobody
c) The government
d) The big tech companies (like Google, Twitter, Microsoft)

6. Do you think that the big tech companies have

a) too much power over your life
b) the right amount of power over your life
c) a little power over your life
d) no effect at all on your life

7. Have you ever used the Internet to get your point of view across to other people?

a) Yes – often
b) Yes – sometimes
c) No

8. Would you like to learn about ways to use the Internet for campaigning or persuading other people?

a) Yes
b) No

9. Do you think that you have any say at all in how the digital world works?

a) Yes, a big say
b) Some say
c) No say

10. Do you think that you should have more say in how the digital world works?

a) Yes
b) No
c) Not bothered
d) Don't know

Youth Jury Post-Session Survey

1. Did you learn anything new today about how the Internet works?

a) Yes, a lot
b) Yes, a little
c) No
d) Don’t know

2. Did you learn anything new today about how the Internet affects your life?

a) Yes, a lot
b) Yes, a little
c) No
d) Don’t know

3. Did you come up with any new ideas today about how the Internet could be made better for people like you?

a) Yes, lots of them
b) Yes, a few
c) No
d) Don’t know

4. On a scale of 1 to 10 (with 1 meaning very little and 10 meaning very much), how much do you agree with the following statements:

i) There should be a trustworthy place for young people to go if they are stressed or upset by things that happen to them online.
ii) It’s OK for websites to pass on details to other businesses or services about people who visit them
iii) The ‘terms and conditions’ for social media sites should be made much clearer to users
iv) It should be made easier for people to remove digital content about themselves that’s embarrassing
v) 12-18 year-olds should have a bigger say on how digital technologies and services are run
vi) The big tech companies are accountable enough to users of digital technologies

5. On a scale of 1 to 10 (with 1 meaning very little and 10 meaning very much), how far do the following statements apply to you:

i) I’m confident that I can influence the way that digital technologies work for young people
ii) Nobody is going to listen to what young people say about the Internet they want
iii) I’ve changed my mind today about how the Internet and other digital technologies should work
iv) When I use digital technologies, I’m in charge of what happens to me
v) When I use digital technologies, I’d like to be more in charge of what happens to me
